# Grandmothers’ care practices in areas of high deprivation of Scotland: the potential for health promotion

**DOI:** 10.1093/heapro/daab104

**Published:** 2021-07-22

**Authors:** Stephanie A Chambers, Fiona Dobbie, Andrew Radley, Neneh Rowa-Dewar

**Affiliations:** 1 School of Social and Political Sciences, Adam Smith Building, 28 Bute Gardens, Glasgow G12 8RS, UK; 2 MRC/CSO Social and Public Health Sciences Unit, University of Glasgow G3 7H3, UK; 3 Usher Institute, University of Edinburgh, Edinburgh EH8 9AG, UK; 4 Directorate of Public Health, NHS Tayside, Dundee DD1 9AG, UK

**Keywords:** grandparents, children, health promotion, assets, family practices

## Abstract

In many families grandparents play an essential role by providing secondary care for grandchildren. The family is a key setting for promoting children’s health; however, studies describing health initiatives with grandparents are rare. Grandparents could play an important role in promoting health for their grandchildren within their families and communities. The aim of this study was to examine the care practices of grandparents in families living in areas of high deprivation, and to consider the extent to which grandparents could be at the centre of health-promoting initiatives for children. A family practices approach was used to examine care practices within the framework of family resource (assets/capitals) use. In-depth interviews were carried out with grandmothers (*n* = 15) and mothers (*n* = 15) living in areas of high deprivation in Scotland. The results are presented as three economies of family living—political, moral and emotional. Grandparent care was described as a form of social capital, central to the wellbeing of the families, and enabled parents to access education and employment. Grandparent care was supported through families’ ability to access cultural amenities and green space (political). Grandparents’ care practices were described as either being responsible or fun (moral). Love appeared to be at the centre of grandparents’ care (emotional). The strengths and weaknesses of this framework were examined in relation to developing initiatives with grandparents. With further development work, grandparents could be the focus of health initiatives with their grandchildren with the support of appropriate policies and resources within their communities.

## Lay Summary

In many families, grandparents help by providing childcare. Children’s health is linked to their family’s overall wellbeing and there have been programmes to improve children’s health within the family. These programmes do not usually include grandparents. In this study, we spoke with 15 grandmothers and 15 mothers living in Scotland about the care that grandparents provide to their grandchildren. They described the different ways in which this care was managed in relation to other aspects of family life, like work. Grandmother care was described in two main ways—as either being responsible or as fun. The main driver of grandparent care was emotional—the love grandmothers held for their grandchildren. We consider the ways in which grandparents, with appropriate support from government at different levels, might help promote health in their grandchildren.

## INTRODUCTION

In many families, grandparents play an essential role by providing secondary care for grandchildren, particularly when state provision of childcare is limited (Di Gessa *et al.*, 2016a). The family is a key setting in which care practices that influence children’s health and wellbeing are situated. These practices, in combination with wider structural influences, can establish habits that may impact on health into adulthood. Grandparent care can be conceptualized as an asset that many families draw from to further family wellbeing. Assets-based approaches seek to draw from a community’s strengths (the resources available at multiple levels) to promote wellbeing, rather than focusing on deficits (Brooks and Kendall, 2013). Alvarez-Dardet *et al.* promote assets-based approaches where structures (Alvarez-Dardet *et al.*, 2015), institutions and processes adapt to enable communities (and families) to be empowered to make changes.

The literature on modifying care practices to improve children’s health has focused on parental roles (Hingle *et al.*, 2010; Thomas *et al.*, 2015; Brown *et al.*, 2016). Parents experiencing socio-economic disadvantage have been more likely to be the focus of health initiatives to modify their care practices (Kumanyika and Grier, 2006, Skouteris *et al.*, 2011; Hesketh and Campbell, 2010). Such initiatives do not take wider family, socio-political and economic contexts into consideration. Similarly, there is limited recognition from academics, policy makers and practitioners of the multiple caregivers who may be involved in caring for children.

In recent years, there have been a number of reviews examining the role of grandparents on various aspects of grandchildren’s health and wellbeing. A systematic review that focused on feeding found mixed results for the role of grandparents, finding that often they provide both healthy and unhealthy food, and encourage both positive and negative eating practices (Marr *et al.*, 2021). Young *et al.*’s review of grandparents’ impact (Young *et al.*, 2018) on grandchildren’s dietary intake found a more negative picture, with the majority of studies indicated that grandparents attitudes and behaviours were a negative influence. Chambers *et al.* looked at a wider range (Chambers *et al.*, 2017) of outcomes in relation to non-communicable disease and found that grandparents were reported in the majority of studies to be a negative influence. In a wide-ranging review of grandparents’ influence on children’s health and development, Sadruddin *et al.* highlight the complexity involved in isolating a (Sadruddin *et al.*, 2019) specific ‘grandparent effect’. They conclude the review by arguing that further research is required examining grandparent care within interpersonal and structural contexts, and propose a conceptual framework for understanding modalities of care, contexts and key outcomes. This conceptual approach was absent in many earlier studies examining grandparents roles, although there are some notable exceptions [e.g. see Eli *et al.* (2016)].

The majority of studies included in the first three reviews cited above report the perspectives of parents only and few presented grandparents’ experiences. More recently, studies have been published that include grandparents’ perspectives in relation to feeding practices, which present the complex interplay of factors present as grandparents care for their grandchildren (Pankhurst *et al.*, 2019; Rogers *et al.*, 2019; Criss *et al.*, 2020).

This brief overview of the literature has suggested that grandparent care has an impact on children’s health and wellbeing, but that current evidence in this area has not adequately engaged in understanding the complexity of interpersonal and wider structural influences on this relationship. This study aimed to understand family wellbeing in the context of grandparent caregiving and wider family resource use. Drawing from 30 in-depth qualitative interviews with mothers and grandmothers, this article examines grandparent care practices among families living in areas of high deprivation. It aims to understand children’s health and wellbeing in the context of grandparent caregiving and wider family resource use. Health and wellbeing in this study includes nutrition, physical activity, screentime, social and emotional health, exposure to substances and exposure to enrichment activities (such as outings and visits to places of interest). With a dearth of evidence for health-promoting initiatives that focus on grandparents’ roles, the discussion considers the extent to which grandparents could be part of an assets-based approach to improving children’s health and wellbeing.

### Theoretical approach

Two complementary frameworks were used to guide the study following data collection: Morgan’s Family Practice Approach (2011) and Bebbington’s five capitals framework (Bebbington, 1999). Morgan approaches the study of family practices (Morgan, 2011), particularly those in vulnerable families, from a solutions-based rather than a problems-based perspective. The complex interactions within families are framed as having the potential to be strengths. Morgan outlines three (Morgan, 2011) economies of family living (political, moral and emotional) which overlap with each other. Political economy is defined as the ways in which families allocate and use resources. Moral economy describes the ways in which families account for their decision making and the values that underpin it, whilst emotional economy involves the role of feelings in families’ accounts about the practices they perform.

In considering the political economy of the family or household, Bebbington classifies five different (Bebbington, 1999) resource types (or capitals/assets): human capital (skills and education); social capital (social networks); cultural capital (ability to access and enjoy cultural amenities); natural capital (ability to access and enjoy green space); and produced capital (income and employment). Bebbington argues that differential access to resources impacts on families’ capabilities and determines the extent to which families are able to reach a degree of wellbeing. Families’ access to resources also impacts their ability to live meaningfully, and to gain the capacity to transform the structures that enable or constrain resource access. The concept of meaningfulness aligns with Morgan’s moral and emotional economies (Morgan, 2011). Bebbington argues that interaction between different resources (Bebbington, 1999) types can result in synergies as well as vulnerabilities. He identifies social capital as central to his framework in relation to other resource types, and defines it as access to a social network. Barker unpicks the social network definition (Barker, 2012) of social capital by describing it as: (i) a contact/relationship; (ii) access to valued resources; (iii) trust or shared norms of obligation, and is the definition used in this study.

## METHODS

### Recruitment

This study received ethical approval from the University of Glasgow’s Research Ethics Committee with data collected throughout 2015. Mothers and grandmothers were recruited via community organizations located in areas of high deprivation in Glasgow and Edinburgh in Scotland. Recognizing the potential for cultural differences in family structures and roles, a community organization who worked specifically with families with an ethnic minority background was included. Organizations were provided with a Participant Information Sheet to support recruitment of parents and grandparents attending their premises on behalf of the research team. Participants mainly attended recreational and educational classes (e.g. art and IT classes) in these locations. Those interested were then informed verbally about the study and had the opportunity to ask questions. Participants gave written consent and were assured of confidentiality and anonymity in the reporting of their data.

### Participants

Thirty participants were recruited: 15 mothers and 15 grandmothers. Although both men and women attended local community organizations, only mothers and grandmothers accepted to participate in the study. Eligibility criteria were families where grandparents were caring for grandchildren under 16 years of age independent of parents on a regular basis (i.e. at least once per month). There were two mother/daughter dyads, and four mother-in-law/daughter-in-law dyads. Other participants were unrelated. Participants lived in areas of high deprivation (defined as being located in the 20% most deprived communities based on the Scottish Index of Multiple Deprivation).

### Procedure

Interviews with eight participants with an ethnic minority background were carried out in participants’ homes at their request. A researcher with the same ethnic background as these participants interviewed them in Urdu and translated transcripts into English. All other interviews were carried out at the premises of community organizations where parents and grandparents visited or attended classes (with the exception of one interview which was carried out on university premises). Two researchers interviewed these participants in English and had the same ethnic background to the participants. Participants were all unknown to researchers before taking part in the study. All researchers had advanced training in qualitative methods at doctoral level. Two grandparents who were close friends chose to be interviewed together. All other interviews were one-on-one and face-to-face. Interview duration ranged from 22 to 55 min. Discussions concentrated on those children whose grandmothers were caring for currently, however, some made reference to previous caring responsibilities for grandchildren who were now adults. Interview questions focused around parents’ and grandparents’ routines with children, and probed questions around children’s health and wellbeing. Participants were asked also about areas of difficulty related to grandparents caring for grandchildren, the benefits to children, parents and grandparents, and intergenerational differences in child rearing (see Supplementary Information 1 and 2 for full list of questions). Interviews were audio-recorded and transcribed verbatim. Non-standard English was translated to standard English where it was necessary to aid clarity for an international audience. Participants received a £15 gift voucher as a thank you for taking part in the study.

### Analysis

Data were subjected to an iterative thematic coding process. First each author read a separate sub-set of the transcripts, and then discussed some of their initial reflections on the data. We then consulted literature around families and health, identifying frameworks relevant to initial emerging themes. Those that theorized family/household resource use appeared to be most closely aligned (Bebbington, 1999, Morgan, 2011). Two authors then read through all transcripts and developed a coding framework (Boyatzis, 1998) based on the data and the literature outlined in the introduction. Each of the four authors coded their assigned transcripts using this framework. A single author reviewed this coding for all transcripts to ensure consistency. Data categorized under each code were then re-read and key themes identified for discussion (Braun and Clarke, 2006).

## RESULTS

Table 1 provides an overview of the participants’ characteristics. The results are presented under the three headings of political, moral and emotional economies. The distribution of these themes across interviews can be seen in Supplementary Information 3. The political economy focuses on families’ resource use. Social capital, as realized through grandparents’ care for grandchildren, was a central resource through which parents were enabled to access human and produced capital (education and income/employment). In addition, participants’ accounts suggested that social capital facilitated families’ ability to access cultural and natural capital, and that affordable access to cultural and natural capital made it easier for grandparents to provide care that was enriching to grandchildren’s wellbeing. Moral economy presents the ways in which participants accounted for the types of care practices grandparents engaged in—as either playing the role of a responsible grandparent or a “fun” grandparent. The emotional economy provides insight into the feelings that were discussed in relation to grandparent care, specifically love.

**Table 1:** Participant characteristics

Participant ID	Role	Grandchildren	Caring arrangements	Age
1	Grandmother	Grandson (4 years)	Cohabiting with grandson. Caring a.m. and weekend evenings	Mother 21 years
3	Mother	(Daughter of Participant 1)		
2	Grandmother	3 grandchildren—caring for granddaughter (2 years) and grandson (7 years). Other granddaughter (6 years)	Overnight care 3 nights per week at grandmother’s with mother and one weekend night alone	Grandmother 59 years
4	Grandmother	Granddaughter (10 years), grandson (8 years)	Cared for granddaughter 6 a.m.–2 p.m. for 3.5 years and grandson before and after school 3 days per week. Grandson overnight care Wed and Sat evenings	Age not discussed
5	Grandmother	3 grandchildren—grandsons (10 and 11 years), granddaughter (10 years)	Previously cared for granddaughter 5 days per week. Cares for grandsons after school and on a Saturday and school holidays	Grandmother 60+ years
6	Grandmother	Granddaughters (2 years and 8 months old)	Cares for 2-year old 2–3 days per week, and 8-month old 1 h 2 days per week	2-year old’s mother 41 years
7	Mother	Daughter (5 years)	Cohabiting with grandparents	Age not discussed
8	Mother	Daughter (11 months)	Previously cohabited with grandparents. Grandparents care for child one day per week	Mother 19 years
9	Mother	Daughter (5 years)	Cohabited with grandparents for first year. Overnight care Sunday–Tuesday	Mother 25 years
10	Grandmother (mother participant 14)	13 grandchildren and 3 great grandchildren, eldest grandchild 26 years and youngest 9 years	Currently cares for granddaughter, often overnight care at the weekend Cared for all previously	Grandmother 69 years
14	Mother (grandmother participant 10)	Son 21 years and daughter 9 years	Grandmother cares for child every second weekend and during summer holidays	
11	Grandmother	3 grandchildren (cares for 15-year old grandson)	Grandmother cohabits with grandson, used to care for him every weekend before he cohabited	Grandmother 70 years
12	Mother	Daughter (1 year)	Cohabits with grandparents Grandparents watched child whilst mother was at school, but now 1 day per week	Mother under 20 years
13	Grandmother	14 grandchildren, 3 great-granddaughters. Currently cares for grandson (7 years) and granddaughter (11 years)	After school 1 day per week and sometimes stay overnight at weekend	Age not discussed
15	Mother (grandmother participant 16)	4 children (20, 16, 11 years and 18 months)	Grandmother cares for 11-year old granddaughter frequently	Mother 35 years Grandmother 66 years
16	Grandmother (Mother participant 15)	12 grandchildren (including 4 great-grandchildren)	Cares for 3 grandchildren and 2 great-grandchildren during school holidays, cared for all grandchildren	
17	Grandmother	Granddaughter (13 years)	Cares for granddaughter after school and at weekends	Age not discussed
18	Grandmother	3 grandchildren—granddaughter (13 years—lives away), grandson (7 years), granddaughter (2 years)	Cares for younger children ad hoc Previously provided care Mon–Fri for grandson before school	Grandmother mentions being older
19	Grandmother	Grandmother, 3 grandchildren—baby (<1 year), grandson (8 years), granddaughter (14 years)	Cares for them after school 3–6 p.m.	Age not discussed
20	Grandmother	5 grandchildren—grandsons (6 and 10 years). Three older grandchildren (20, 16, 15 years)	After school care 5 days per week. Overnight care Saturday	Age not discussed
21	Mother	Daughter (6 years), Son (2 years)	Overnight care weekends and ad hoc	Grandmother 65 years Grandfather 67 years
22	Mother	Daughter (8 years) Son (2 years) Currently pregnant	Grandmother provides care early mornings	Age not discussed
23	Grandmother	2 grandsons (8 and 12 years)	Grandmother cohabits with grandsons	Age not discussed
24	Mother	Daughter-in-law of P23		
25	Grandmother	Granddaughter aged 4 years	Grandmother provides ad hoc childcare	Age not discussed
26	Mother	Daughter-in-law of P25		
27	Grandmother	3 grandchildren, grandson (7 years), granddaughters (9 and 17 years), and 4 non-cohabiting grandchildren	Grandmother cohabits with 3 grandchildren	Age not discussed
28	Mother	Daughter-in-law of P27		
29	Grandmother	3 grandchildren—(6 and 7 years—no age given for third grandchild)	Grandmother cohabits with 1 grandchild	Age not discussed
30	Mother	Daughter-in-law of P29		

### Political economy—the interaction of social capital and family resource use

Participants’ descriptions of grandparent care were in line with the definition of social capital outlined earlier, particularly as access to valued resources. In discussing the political economy of the family, participants valued grandparent care as a way to access work or further study. Mothers described relying on grandparent care due to necessity, particularly when they were working in insecure sectors that paid minimum wage or non-standard hours.

I wouldn’t be able to do the course that I was doing if my mum didn’t do it [provide care] because especially like with the shifts and stuff, and because it varies from week to week and day to day. So I wouldn’t be able to take them to a child-minder. Participant 21, Mother, 2 children, currently pregnant

For the participants, grandparent care had mitigated the effects of a perceived lack of policy support for those requiring childcare whilst working in low status jobs. One participant explained that after separating from her daughter’s father she needed to take on low paid employment and relied on her mother for childcare. Over time, this support had enabled her to find higher paid work and to achieve a higher standard of living.

I think for a lot of people if they didn’t have family support then they would be doomed…we struggle more. … Do I want to be sitting in a job that’s going be paying pennies, basic wage, or do I get myself out there and do something that I want to do that’s going to bring in more money? Participant 14, Mother, 2 children

Some participants explained that at the time of interview state funded childcare for 2–4 year olds only covered 15 hours per week, often with limited opportunity to choose when these hours were provided, and therefore they did not perceive that it facilitated parental employment. An additional issue was raised by two grandmothers. They believed that government had pressurized young families in recent years through the welfare system by limiting parents’ welfare benefits if they were not in employment. In the example below, this grandmother outlines issues within the welfare system, but at the same time stressed her daughter’s work ethic.

They’ve [Parents] got no other option nowadays to go out and work. If they don’t go look for work they’re not getting money off the Buroo - the Social [the Department for Work and Pensions]. And if they miss appointments their money’s stopped. My daughter prefers working really than sitting about. So, I said to her, ‘Well, get a job and I’ll look after her.’ Participant 10, grandmother, 13 grandchildren

Grandmothers described relying on locally funded amenities to provide access to cultural and natural capital in the form of activities for children. This supported them to provide care that was health enhancing and enriching often promoting physical activity at low cost. Local parks were the main places that grandmothers described taking children to outside of the home. Younger children had the opportunity to play on playground equipment, whilst older children played sports.

I’ve took them down to a wee zoo in the park, they’ve got a wee farm thing…I’ll walk up the loch [Scottish lake] with them sometimes along to the loch, just take a wee walk about the loch. The wee one doesn’t like it ‘cause he’s got to use his legs (laughs) you see. My other grandkids, they’re that used to cars. You know everybody’s got a car now. Participant 13, Grandmother, 14 grandchildren, 3 great-grandchildren

Other local amenities described where cultural capital was accessed were municipal-owned swimming pools and ‘soft play’ gyms offering free or affordable sessions. Spaces that offered free entry, such as art galleries and museums, subsidized through local government, were also highlighted as places where grandparents visited with grandchildren. One grandmother covered a range of these amenities when she said:

Every single time he [grandson] comes, ‘Oh you’re the best granny in the world’ ‘cause I take him everywhere…We go to the park, go to the art galleries, go to the cinema, go to the swimming quite a lot’. Participant 4, Grandmother, 2 grandchildren

### Moral economy—accounting for care practices and grandchildren’s health and wellbeing

Participants described the ways in which grandparents performed care practices likely to impact on grandchildren’s health and wellbeing. Grandparents provided food, made decisions about how grandchildren should spend their time in recreational activities and about the volume of sleep children required. Grandmothers positioned themselves as providing either responsible caregiving with traditional values and rules, or as caregiving focused around fun, ensuring their grandchildren’s happiness and enjoyment. Mothers’ descriptions of grandparents were generally aligned with these two portrayals, with some mothers also discussing grandfathers and their care practices. A small number of mothers and grandmothers described tensions that had arisen within the family due to differences in values around caregiving.

#### Responsible caregiving practices

Some grandmothers provided an authoritative and traditional approach, carrying out practices perceived to enhance their grandchildren’s wellbeing in a way that aligned with recommendations around diet, physical activity and sleep. For example, one grandmother was highly critical of her daughter’s parenting. The grandmother criticized the food her daughter provided, the fact that she smoked around her grandson, and the lack of discipline her daughter instilled. She contrasted this with her approach, which included disciplining her grandson, providing healthy lunches and snacks for him, and spending time reading with him. This grandmother said:

If he [grandson] is doing things wrong in the house and I tell him off, she’ll say, ‘It’s my wean [child].’ I’ll say, ‘Well, why are you sitting there then and letting me run after him?’ It causes a lot of tension. Participant 1, Grandmother, 1 grandchild

Other grandmothers expressed pride that they provided grandchildren with home cooked meals. Home-cooked meals were valued for their health properties, but also as a demonstration of grandparents’ love for their grandchildren.

Probably a wee bit more home-cooked in granny and granda’s [grandfather]. Definitely. As I say, I’m a wee bit more about convenience, whereas my dad’s more about - he’ll prepare. He’s a preparer of food. Participant 20, Mother, 2 children

Participants with ethnic minority backgrounds highlighted that grandmothers’ co-residence with grandchildren resulted in children eating more traditional foods, such as curries, rather than processed foods.

Some grandparents described their remit as extending to discretionary foods as well as meals, and they said that they limited grandchildren’s consumption of foods considered less healthy. They described instilling various rules to manage this consumption, such as having to ask permission before taking something, or limiting the quantity of discretionary foods they consumed.

I always do a pudding on a Sunday, a dessert. And the wee one thought when he comes to me during the week he should get dessert ‘cause he got it on a Sunday from me. And I said, “No, it doesn’t work, we have fruit during the week.” Participant 13, Grandmother, 14 grandchildren, 3 great-grandchildren

Grandmothers’ portrayals of performing these responsible caregiving practices were also demonstrated in relation to children’s leisure time activities. Grandparents valued outdoor activities where children could play, indoor activities where they could be physically active, or cultural activities. Engaging in these valued activities was contrasted with excessive screen time, which a number of grandparents said they limited when they cared for grandchildren.

He’s [grandson] got an iPad, he’s got an iPhone. He’s had that from when he was a wee boy. He’s got everything because he’s the only one. He’s quite spoiled. So we tend to take it from him… because he would sit there all day with it. “Come on, time to get out.” Participant 4, Grandmother, 2 grandchildren

When asked to discuss smoking and drinking alcohol around their grandchildren, grandmothers again highlighted the ways in which they acted responsibly. One grandmother described the changes she made in anticipation of becoming a grandparent by stopping smoking ahead of her grandchild’s birth:

I did smoke but I stopped ten years ago…I stopped when my daughter-in-law was pregnant with my granddaughter. I stopped then because obviously there was a grandchild coming. Participant 4, Grandmother, 2 grandchildren

Even where grandmothers described engaging in practices that were not recommended in relation to children and smoking, such as smoking in the same house as grandchildren, participants positioned these acts within the responsible grandparent narrative. They described modifying their usual practices to mitigate the impact of children’s exposure.

I go out in the landing, but usually I smoke in the living room but if [my granddaughter’s] in the room I open a window, you know…I don’t drink, just the smoking. Participant 10, Grandmother, 13 grandchildren

Two examples were provided by one grandmother of her care for her grandchildren’s wellbeing extending beyond direct caregiving. In the first instance, she described intervening to alleviate occasions when her daughter experienced food insecurity.

I don’t mind feeding my grandkids. I mean, I’ve seen my daughter coming up and saying, ‘Look, I’ve not really got anything in the house’, ‘cause she’s struggling by the time she pays bills and this and that and the next thing. That doesn’t bother me. I feed them. I wouldn’t like to live when you see them doing without. Participant 13, Grandmother, 14 grandchildren, 3 great-grandchildren

In the second instance, she said she confronted her son about his alcohol consumption as she was concerned that it could lead to the breakup of his family.

I think they’ve [grandchildren] seen their daddy with a drink and I have said to him, not a nice man when he’s drunk, just like that. And he isn’t, he just turns on drink. Drink’s not for him. And I said, “See if you carry on like that, you’re going to lose your weans [children] and your wife, carrying on the way you are.” Participant 13, Grandmother, 14 grandchildren, 3 great-grandchildren

This grandmother positioned herself as going above and beyond being a responsible caregiver to their grandchildren, and instead described the matriarchal role that she fulfilled within her family.

#### Fun caregiving practices

Although participants presented multiple examples of grandparents acting ‘responsibly’ in relation to performing caregiving practices, many grandmothers also spoke at length about the fun experienced when performing caregiving practices. At times this overlapped with their role as a ‘responsible’ grandparent, for example, in relation to encouraging children to take part in physical activity. They detailed the long list of places where they would take their grandchildren to spend time, including walking, visiting parks, supervising outdoor play and accompanying children to swimming pools or indoor playground locations. Some grandparents had gardens where children could play safely.

When it’s the summer holidays we go to the park, we go swimming, and sometimes my friend and I who stays up the flats…we sit outside. We’ve got our wee chairs, our folding chairs, we take them downstairs and the kids play on their bikes or football. Participant 5, Grandmother, 3 grandchildren

However, there were other ways in which grandparents described caring for grandchildren that were not in line with health recommendations. In some cases, mothers wished that grandparents would discipline their children appropriately whilst caring for them. They believed that this would reduce the likelihood of grandchildren misbehaving.

I think I’d discipline them probably a wee bit more than granny and granda [grandfather]. I don’t dead [really] disagree with them, but what I try and say to them is, “Don’t let them away with things like this, ‘cause I wouldn’t”. Participant 20, Mother, 2 children

Some grandparents appeared to embrace their role as a treat provider, spoiling their grandchildren and providing them with opportunities to eat high sugar or fat snacks or takeaway meals. In the examples below, grandmothers presented these less healthy practices within the context of grandchildren enjoying their grandmothers’ homes as welcoming settings where they engaged in a range of fun activities.

Researcher: Do you give them treats?

Yes…Well there’s stacks in the house, I just keep buying and putting it in the cupboard. So they’ll say, “Gran, can I get a bit of chocolate?” So I allow them a bit of chocolate. Participant 4, grandmother, 2 grandchildren

#### Emotional economy—grandparent care as an exchange of love within families

When discussing the performance of care practices, it was clear that the meaning of these activities went beyond the facilitation of parents’ work, and that the real exchange was not one of childcare, but of love. Whether grandparents’ caregiving practices were presented as ‘responsible’ or ‘fun’, all were framed within a wider context of practicing love for their grandchildren. Grandparents’ caregiving practices served to build and sustain relationships between their grandchildren, but also their children. Morgan has argued that emotion (Morgan, 2011) has not been central to theories of social behaviour; however, for the participants in this study, practicing love was a means through which families were able to access resources. Grandparents hoped to facilitate economic opportunities for their children, and in doing so they gained the benefit of a close relationship with their grandchildren. Both mothers and grandmothers said providing care kept grandparents young, made them laugh and improved their mental health.

Grandmothers who now had adult grandchildren were proud of the bond that developed when they were children, and this had been sustained long term. Grandmother care was therefore an investment by families. The relationships built were an opportunity for them to share what that they had learned in their own lives, and to gain new knowledge from their grandchildren. Grandmothers described the love shared with their grandchildren as being substantially different to that shared with their own children.

P22: Unconditional love. It’s unconditional love but…

*P 19: Oh yes. I think there’s more enjoyment.*

P22: It’s something… you’d need to be a gran to understand. It’s not that you love your grandkids any more than you love your (*P19: oh no*) children, but it just… it feels like a different kind of love.

*P19: … you don’t have the same responsibility as you had with your own. You enjoy it more, it’s more relaxed.* Participant 22, Grandmother, 3 grandchildren; Participant 19, Grandmother, 2 grandchildren

One grandmother expressed concern that her co-resident 15-year old grandson was distancing himself from her care, and she identified a consequence of this being his engagement in unhealthy practices, such as smoking. She described her aspiration for him to experience life beyond their council estate and her hope that her contribution to his care would endure to re-direct his life onto a more positive path.

I would like him [grandson] to see that this [estate] is not the edge of the world, or if you leave [this estate] you’ll not fall off the edge of the world. Know what I mean?…I would just like him to grow, just to, just be a good person, not to do anything bad…To be happy, get a job, not do anything bad, be considerate of others, have good morals and things like that. That’s what I would like for him*.* Participant 11, Grandmother, 3 grandchildren

The potential health benefits for grandparents in the exchange were discussed by two mothers whose fathers had suffered from depression. They explained that the birth of their children had given the grandfathers a reason to keep going, and had improved the ability of the family to remain resilient in the face of adversity.

My dad suffered with depression for…10 years, and I feel when she [daughter] came along it was like a whole new lease of life for him and he’s got this wee person to focus on so it kind of – he’s got to keep well for her. Participant 9, Mother, 1 child.

Although caring for grandchildren was framed by most participants as a positive exchange in relation to grandparents’ health, a small number of grandmothers described their inability to keep up with grandchildren due to health issues and tiredness.

This year I'm not very well, I've got a bit of breathlessness and they tell me there's nothing wrong with me so I'm thinking it's just my weight so it's up to me to sort it. Participant 5, grandmother, 3 grandchildren

Mothers also noted their concern with the impact that caring might have on grandparents’ health. Many of the participants were aware that realism was required in terms of grandparents’ capacity to provide care, given that some of them were in their 60s and 70s.

When they’ve [grandparents] got them [grandchildren] all weekend I can see it in them on a Sunday, they’re knackered [exhausted]. I can see my mum and dad it’s just like they cannot wait for them to go away. Participant 20, Mother, 2 children

Fathers and grandfathers were largely absent in grandmothers’ and mothers’ accounts of childcare practices. Two grandmothers said that their husbands had died, other participants said that grandfathers had long-term illnesses, or that grandmothers and grandfathers were no longer in a relationship. Intergenerational bonds appeared to be particularly strong in families where fathers were not involved in the care of their children. Many participants expressed hostility in the interviews towards children’s fathers. In some situations the breakdown in relationship between the mother and father had extended to the wider family. Some grandmothers were hostile towards paternal grandparents, and angry that they were either unwilling, or argued they were not appropriate people, to be involved in the lives of their grandchildren.

Not that my daughter’s with her [granddaughter’s] dad. You know, they’ve not been together for years. But he doesn’t maintain [provide financial support] or nothing, which I think is disgraceful, really disgraceful. And her other granny, she never phones the kid or nothing. And when I see her I go, ‘Call yourself a granny? You’re bang out of order.’ Participant 10, Grandmother, 13 grandchildren

Where fathers were absent, grandmothers (and grandfathers where present) played the role of a second parent to a grandchild. Their care provided not only the resource of safe, reliable and affordable childcare, but also a loving and caring environment and role models for children that may otherwise have been absent in their lives. An example was provided of a grandfather fulfilling this role by one mother:

I think because her dad, he’s not been very, very good in her life, because my dad has been more of a father figure for her. Participant 9, Mother, 1 child

## DISCUSSION

### Grandparents’ roles

This study found that grandparents played a substantial role in supporting their families’ health and wellbeing. Grandparents were a source of social capital and their care enabled families to access other valued resource types. Grandparent care allowed parents to work, but the care was sustained by grandparents’ love and enjoyment of the time they spent with grandchildren. Beyond care, however, many participants described the regulatory role which grandmothers fulfilled within the family. This included providing advice to parents and/or attempting to meet children’s needs when there was a perceived area of concern. This included discipline, food, screentime, physical activity or absence of another secondary caregiver. It was clear that grandparents’ involvement in family life was not on the periphery, but often at its heart. The findings of the study suggest therefore that family-based interventions to promote health are likely to be limited if they do not include grandparents, at least in countries where grandparents feel that this is an important part of their role. For example, in Norway, 80% of grandmothers strongly agree that it is their duty to help a grandchild in need, compared with only 30% of grandmothers from the Netherlands (Herlofson and Hagestad, 2012). There are further considerations raised from the literature in terms of the culturally situated roles that grandparents might play. For example in western countries, that tend to be more individualist in terms of the focus on the nuclear family, grandparents may not play a prominent role in family life. In contrast, in more collectivist countries, they may be more likely to have a matriarchal or patriarchal role and this needs to be considered in intervention development design (Herlofson and Hagestad, 2012). Despite being located in the UK, the families in this study, appeared to align more closely with a more collectivist approach, possibly due to the constraints of work, income and childcare that they highlighted.

There were however indications of potential barriers to including grandparents that would need to be considered in future intervention design. Participants discussed a wide range of health conditions which reduced grandparents’ capacity to care for their grandchildren. There are mixed findings in the literature as to whether caring for grandchildren negatively impacts grandparents’ health further. Many studies have indicated that grandparents who take on a primary carer role for their grandchildren are more likely to experience poorer health outcomes (Hayslip Jr *et al.*, 2019). In terms of secondary caregiving, Di Gessa *et al.* found in a large longitudinal (Di Gessa *et al.*, 2016b) study of grandparents across Europe that grandmothers who provide both intensive and non-intensive childcare were more likely than grandmothers providing no childcare to have better physical health. Similar results were found for Chinese grandparents (Zhou *et al.*, 2017), yet Ates reports that positive results (Ates, 2017) are likely to be overestimated. It should be remembered however that interventions to improve children’s health and wellbeing do not need to be linked specifically with a substantial caring load, but could be targeted more on perceived capacity to participate fully in specific intergenerational activities and practices.

An additional barrier to intergenerational interventions were the tensions reported by some participants around divergent approaches to caring practices. The possibility of conflict between generations has been recognized previously in relation to grandparent care (May *et al.*, 2012, Sivak, 2018). Caution is therefore necessary around expectations and understandings of the factors that might impact positively or negatively on children and families’ health and wellbeing. These are likely to differ for some families between parents and grandparents. Linked to this is that a few participants indicated that some riskier health behaviours served other positive functions. For example, in some families, provision of less healthy foods served as a means through which grandchildren and grandparents strengthened their bond. This was also found in a recent study of grandparents in Denmark and New Zealand where time spent with grandchildren was discussed as a time for treats (O’Donohoe *et al.*, 2021). Interventions would seek to avoid negative unintended consequences in terms of the impact they have on relationships.

### Potential of interventions with grandparents

Previous interventions that have included grandparents have focused on grandparents who are primary caregivers and developing and enhancing their parenting skills (Chan *et al.*, 2019). Outcomes for children have typically concentrated on their social and emotional health and wellbeing, rather than physical health. The results from a meta-analysis indicate that these interventions have the potential for success, which suggests that interventions that focus more on other health outcomes might also be effective (Chan *et al.* 2019). Fruhauf *et al.* call for interventions with grandparents (Fruhauf *et al.*, 2020) to take a strengths-based approach, drawing from the range of internal and external assets held by families and communities. They stress that interventions need to engage with outcomes for both grandparents and grandchildren, and should not be one way in their focus. Mansson found that grandparents identified (Mansson, 2016) some of the greatest joy in their relationship with their grandchildren through shared activities, and teaching and learning. Similarly in a qualitative study of Czech grandmothers and mothers, the relationship between grandmothers and grandchildren was fostered through shared activities and interests (Marhankova, 2019). Interventions that build on this foundation of these two different generations spending time together, and engaging in mutually appreciated, health-promoting activities are likely to have greatest potential.

### Public investment in extended families and communities/structural support

Bebbington argues that it is ‘important to have a clear sense of the most important assets for different people in different places in order to identify the most useful (and most damaging) sorts of public investment in such areas.’ [(Bebbington, 1999), pp. 2031–2032]. Our analysis has provided some direction when considering public investment to support a role for grandparents in promoting children’s long-term health and wellbeing.

Parents’ employment opportunities in areas of socio-economic disadvantage were often constrained by low wages, unsociable hours and insecure contracts. Grandparent care enabled parents to manage in difficult conditions masking the need for public infrastructure that supported families. The sustainability of this social capital was supported by the amenities available in the local area that provided affordable access to natural and cultural capital. It allowed grandparents to provide care that was enriching to their grandchildren.

Examples were presented of local government providing support for participants to access natural and cultural capital, such as park and swimming pool facilities. Engaging in practices that allowed participants to access these resources supported grandparents in the provision of care for their children, strengthening social capital and promoting wellbeing. It must be remembered, however, that the participants in this study lived in large urban areas with a substantial range of amenities, and that families living in more rural or remote areas, are unlikely to have the same levels of access. What these examples suggest, however, is that there is a need for public infrastructure that can support extended families.

Some UK-based examples of recent policy change that could support grandparent care, without overburdening families, are increased state-funded childcare hours (UK Government, 2021a), and changes to the national insurance system (UK Government, 2021c) and workplace rights that allow all employees the opportunity to request flexible working (UK Government, 2021b). These policies help to reduce barriers to access to human and produced capital. With policies like this in place, grandparents are likely to have greater capacity to be part of initiatives that promote health in their grandchildren.

### Strengths and limitations

This study is one of the few to engage theoretically with the potential for grandparents to play a role in health promotion for their grandchildren when considering families’ wider resource use. It provides perspectives from two generations, rather than parents only, and has identified areas where greater support from the state may be required before assets-based approaches to promote children’s health and wellbeing can be put in place.

In the families we interviewed, grandparents were generally involved in childcare in a substantial way. From these interviews, data saturation appeared to be reached with findings repeated towards the final interviews. Findings and recommendations may not be relevant to families or cultures where grandparents are less involved in childcare or there are different expectations around grandparents’ roles. We recruited families through community organizations, indicating that participants were likely to have greater ability to access cultural capital than many other families. Our findings also reflect a gendered account of care practices as we only recruited mothers and grandmothers to the study, with resource limitations preventing additional data collection with fathers and grandfathers. Just under half of the participants were from the same families (*n* = 14), however, further insight may have been provided had all parents and grandparents belonged to the same families.

## CONCLUSION

The results from this study show how integral grandparents are to families living in areas of high deprivation, health initiatives with grandparents providing secondary care at their centre are rare. The richness from the analyzed interviews highlights that any initiatives would need to take into account the ways in which grandparent care interacts with other resource use, including the need for the state to have a strong role. The results suggest that the social capital that is accessed by parents through grandparent care could form a strong basis for health-promoting initiatives. However, vulnerabilities in this framework highlight that wider structural supports are required to sustain interventions with grandparents and grandchildren.

## SUPPLEMENTARY MATERIAL

Supplementary material is available at *Health Promotion International* online.

## ETHICS INFORMATION

This study was approved by the College of Social Sciences Research Ethics Committee at the University of Glasgow (UREC Reference: 400140106)

## Supplementary Material

daab104_Supplementary_Tables
