# Supplementary material for: Grandmothers’ care practices in areas of high deprivation of Scotland: the potential for health promotion
Source: Health Promot Int. 2021 Jul 22;37(2):daab104. doi: 10.1093/heapro/daab104 (PMC9067444; doi:10.1093/heapro/daab104)
Supplement: daab104_Supplementary_Tables [file daab104_Supplementary_Tables.docx]

**Supplementary Information 1 - Parent interview** **schedule**

How many children do you have, and what ages are they?

Can you tell me about how your parent(s) help you by looking after your children? (probe – how often, days of week, time).

For how long have your parents helped you in this way?

How has this support changed from when your children were younger? And how do you think this support will change as your children get older?

**Caring routines**

Can you tell me what a typical weekday is like for your family when you look after the children?

Can you tell me what a typical weekend day is like for your family when you look after your children?

[for both questions, probe about transport, food, work, school, nursery, hobbies etc]

**Benefits**

Can you describe the main benefits that receiving childcare support from your parent(s) has brought to your family?

Can you describe the main benefits to your parent(s) in providing your family with childcare support?

**Challenges**

Can you describe the main challenges that receiving childcare support from your parent(s) has brought to your family?

Can you describe the main challenges to your parent(s) in providing your family with childcare support?

When your child(ren) are with your parent(s), what are the main ways that their routines change from when they are at home, or are being cared for by you?

Are there any ways in which you would like your parent(s) to care for your child(ren) that they do not currently? (probe food, exercise/play, hobbies, smoking, sun exposure)

Have you ever spoken with your parent(s) about the way in which they care for your child(ren)?

In what ways is your parent different with your child than they were when bringing up you (and your siblings)?

[If families report tensions around issues relating to childcare] Are there any ways in which you feel you could be better supported to raise issues with your parent about childcare?

**Concluding questions**

Is there anything else that we haven’t spoken about that you think it would be important to know about?

Do you have any other questions for me now based on what I have been asking?

**Supplementary Information – 2 - Grandparent interview** **schedule**

[for all relevant questions, probe if there are differences based on different grandchildren]

**Introduction to study and introductory questions**

Study introduction and opportunity for participants to ask questions.

How many grandchildren do you have?

How old are they?

**Care provided**

When do you look after your grandchildren (probe – how often, days of week, time and what parents are doing during that time)?

Do you look after them on your own?

For how long have you helped out in this way?

How has this support changed from when your grandchildren were younger?

**Caring routines**

Can you tell me what a typical week day is like when you look after your grandchild/ren?

What would you do during the week if you weren’t looking after them?

[probe about transport, meals, drinks, snacks, smoking if they smoke, drinking, work, school, nursery, leisure, work etc. Probe whether the grandparent has certain rules in their house that the children need to stick to]

How is it different at the weekend?

Is there anything else that you do during your care for them that you think it would be important to know about?

**Benefits**

Can you describe the main benefits to your children in providing them with childcare support?

Do your children ever tell you what they like best about you caring for their child(ren)?

What are the main benefits to your grandchildren?

Can you describe the main benefits to you in providing childcare support?

**Challenges**

Can you describe the main challenges for you in providing childcare support?

Some parents have very set ideas about the way they want to bring up their children. Has your child ever spoken with you about the way they would like you to care for your grandchild(ren)? (probe smoking, leisure, food, sun exposure)

[As a further probe] Would your son or daughter ever raise any issues about the way that you care for their child that they are unhappy with?

When your grandchildren are with you, what are the main ways that their routines change from when they are at home, or are being cared for by their parents?

(probe about transport, food, smoking if they smoke, work, school, nursery, hobbies etc. Probe about parental rules that might be different)

Are there any ways in which you would like to care for your grandchild(ren) that you feel you are not able to do currently?

What do your children say if you raise these issues about that?

[If the grandchildren are older] Have you every spoken with your grandchildren about that?

What might be the best ways for families to approach differences in opinion around child care?

Are there any organisations out there that you know of or have heard about that could help with this? Is that something that you would be interested in if support was available?

**Changes in bringing up children**

How do you feel bringing up children has changed from when you brought up your own children?

Do you feel that it’s better or worse now?

Can you remember any health advice you got when you were bringing up your own children?

How do you think that has changed over the years?

How do you think the support you provide will change as your grandchildren get older?

**Concluding questions**

Is there anything else that we haven’t spoken about that you think it would be important to know about?

Do you have any other questions for me now based on what I have been asking?

**Supplementary Information 3 – Theme distribution across interviews**

| **Participant ID**  **Role:**  **Grandchildren & ages**  **Caring arrangements (social capital)** | **Political Economy (human and produced capital (H&PC), natural and cultural capital (N&CC))** | **Moral economy** | **Emotional economy (Other parent involvement; Parenting tensions; Love and care; Grandparent health)** | **Areas of health discussed that grandparents involved with during care grouped by positive/negative health risks** |
| --- | --- | --- | --- | --- |
| **Participant 1**  Grandmother  Grandson (4 years)  Cohabiting with grandson and daughter - caring am and weekend evenings  **Participant 3**  Mother (Daughter of Participant 1) | **H&PC**  Mother and grandmother  not currently working  Father works away  **N&CC**  Parks  Community cafe | Responsible caregiving  Fun caregiving | **Parenting**  Parents in relationship but not cohabiting; Do not speak to paternal grandmother; Grandparents separated but grandfather has contact  **Tensions**  Grandmother critical of mother’s parenting; Grandmother provides advice to mother  **Love & care**  Wants daughter to live full life; Provides focused attention; Enjoyment; Instils routine | **Positive**  Outdoor exercise/play  Routine  Focused attention |
| **Participant 2**  Grandmother  3 grandchildren (caring for granddaughter (2 years) & grandson (7 years)). Other granddaughter (6 years)  Overnight care 3 nights per week with grandmother  with mother & one weekend night alone | **H&PC**  Mother’s working status not disclosed  Grandmother  not working  Financial difficulties described  **N&CC**  Accessing parks | Responsible caregiving  Fun caregiving | **Parenting**  Father does not cohabit and contact with children not discussed; Grandfather died but grandparents were divorced  **Tensions**  Grandmother critical of mother’s parenting  **Love & care**  Patience; Love of the children  **Grandparent health**  Backache; Asthma; Not fit to take grandchildren on holiday | **Positive**  Outdoor exercise/play  Reading and singing  Limits screentime  Patient caregiver  **Negative**  Provides foods HFSS |
| **Participant 4**  Grandmother  2 grandchildren - granddaughter (10 years); grandson (8 years)  Cared for granddaughter 6am-2pm for 3.5 years  Cares for grandson before & after school 3 days per week & overnight care Wed and Sat | **H&PC**  Parents of grandson work. Informal care avoids father having to work more hours as mother can work  Grandmother  not working  **N&CC**  Parks  Seaside access  Swimming pool  Art gallery | Responsible caregiving  Fun caregiving | **Parenting**  Fathers cohabits; Grandmother lives alone  **Love & care**  Closeness/bond through acts of care; Listens to child; Secure caregiver; Caring when sick; Greater leniency; Child expressions of love | **Positive**  Outdoor exercise/play  Enrichment activities  Toothbrushing  Gave up smoking for grandchild  Eating together  Homemade meals  Encourages fruit intake  Limits screentime  **Negative**  Provides foods HFSS |
| **Participant 5**  Grandmother  2 grandsons (10 & 11 years), granddaughter (10 years)  Cared for granddaughter 5 days per week previously  Cares for grandsons after school, Sat, school holidays | **H&PC**  Mother working  Grandmother  not working  Food costs  Childcare prohibitively expensive  **N&CC**  Park  Swimming pool | Responsible caregiving  Fun caregiving | **Parenting**  Grandsons’ parents separated; Granddaughter’s father cohabits; Grandparents separated but grandfather involved in care  **Tensions**  Conflict over extent of grandmother  Care; Mother perceived as too harsh  Grandmother reflected on domestic conflict; Concerned with child discipline  **Love & care**  Love described; Laughter; Caring when sick; Putting grandchildren first  **Grandparent Health**  Grandmother’s breathlessness /excess weight | **Positive**  Outdoor exercise/play  Enrichment activities  Swimming  Homemade meals  Encourages vegetable intake  Limits screentime  **Negative**  Provides foods HFSS |
| **Participant 6**  Grandmother  2 granddaughters (2 years and 8 months)  Cares for 2 year old 2-3 days per week, and 8 month old 1 hour two days per week | **H&PC**  Father works full time, Mother works part time  Grandmother not working  Childcare prohibitively expensive  Childcare prohibitively expensive  **N&CC**  Parks | Responsible caregiving  Fun caregiving | **Parenting**  Fathers cohabiting; Grandfather helps with childcare  **Love & care**  Play; Secure caregiver; Greater leniency  **Grandparent Health**  Caring keeps her youthful/fit | **Positive**  Outdoor exercise/play  Role models healthy eating  Sticks to healthy foods provided by mother  Sun protection  **Negative**  Occasional provision of foods HFSS |
| **Participant 7**  Mother  Daughter (5 years)  Cohabiting with grandparents | **H&PC**  Mother at college – grandparent care opportunity to have career and future  Grandmother  not working  **N&CC**  Park | Responsible caregiving  Fun caregiving | **Parenting**  Father involved in child’s life but not cohabiting; Grandfather very involved  **Tensions**  Tension over routines and ex-partner and other grandparents, other grandchildren  **Love & care**  Identified different love shared between grandchildren & grandparents; Second chance at parenting; Grandparents supported mother through depression; Love described; Happiness; Family unit; Play  **Grandparent Health**  Grandmother’s backache; Keeps them young; Childcare helped grandfather recover from illness | **Positive**  Play  Homemade meals  Soft play/dancing  Walking  Neither grandparent drinks alcohol  Help with weaning  **Negative**  Grandfather smokes in home |
| **Participant 8**  Mother  Daughter (11 months)  Previously cohabited with grandparents. Grandparents provide care one day per week | **H&PC**  Father working  Mother unable to work as childcare prohibitively expensive  Grandparents working  **N&CC**  Park  Beach  Local baby groups | Not discussed | **Parenting**  Father cohabits; Grandfather involved in childcare  **Tensions**  Tensions with grandparents around routine and smoking  **Grandparent Health**  Keeps grandparents busy | **Positive**  Encourage outdoor play  **Negative**  Provide chocolate  Grandfather smokes in home |
| **Participant 9**  Mother  Daughter (5 years)  Previously cohabited with grandparents. Grandparents provide overnight care Sun-Tues | **H&PC**  Mother works part time  **N&CC**  Community centre activities | Responsible caregiving  Fun caregiving | **Parenting**  Father not involved. Grandfather involved in childcare  **Tensions**  Previous conflict with paternal Grandparents over routine  **Love & care**  Supported mother when unexpectedly pregnant; Secure caregivers; Relaxed care; Enjoyment; Instil routine  **Grandparent Health**  Childcare helped grandfather through depression | **Positive**  Outdoor play/exercise  Routine  Homemade/fresh food  Limits snacking  Grandparents do not drink alcohol  Role model healthy eating  **Negative**  Provides foods HFSS |
| **Participant 10**  Grandmother  (Mother of participant 14)  13 Grandchildren (eldest 26 years; youngest 9 years) and 3 great grandchildren  Looked after all of them  Currently caring for granddaughter (9 years) often overnights at the weekend  **Participant 14**  Mother (Grandmother  Participant 10)  Children: Son (21 years) & Daughter (9 years)  Grandmother cares for granddaughter every second weekend & during summer holidays | **H&PC**  Mother’s previous shift-work; M currently works 16 hours p/w – shift work – unable to find FT, well-paid work.  Grandmother not working  Ensuring children have enough food when parent struggling financially  Childcare prohibitively expensive  Family break up caused financial difficulties  Benefits system forcing parents to work  **N&CC**  Park | Responsible caregiving  Fun caregiving | **Parenting**  Father and paternal grandparents not involved in care. Grandfather died.  **Tensions**  Similar views on discipline as M; Wants grandmother to discipline more  Thinks grandparents do not give grandchildren opportunity to speak  **Love & care**  Bond between grandchildren & grandmother  **Enjoyment**  Mutual enjoyment  **Grandparent Health**  Grandfather suffered from alcoholism  Care keeps grandmother young  Mother concerned about grandmother’s age and health | **Positive**  Sufficient sleep  Breakfast  Outdoor exercise/play  Homemade meals  Provides vegetables  Routine  Homemade food  **Negative**  Believes in physical chastisement  Provides foods HFSS  Smoking |
| **Participant 11**  Grandmother  3 grandchildren including grandson (15 years)  Previously cared for grandson every weekend.  Cohabits with grandson currently | **H&PC**  Grandmother not working  Mother working Full time as hairdresser  **N&CC**  Swimming pool  Park when younger | Responsible care giving  Fun care giving | **Parenting**  Father not involved  Grandfather died  **Tensions**  Mother has told grandmother she interferes  **Love & care**  Worries for grandson’s wellbeing/ success; role modelling health behaviours and morals; wish for an enduring relationship; mutual exchange of learning and love  **Grandparent Health**  Rheumatoid arthritis | **Positive**  Encourages vegetable intake  Outdoor exercise/play (previously)  Swimming (previously)  **Negative**  Provides foods HFSS |
| **Participant 12**  Mother  Daughter (1 year)  Cohabiting with grandparents.  Grandparents cared whilst mother at school, now only on a Tue | **H&PC**  Mother not working  Grandparents work status unknown  Grandmother was given carers’ allowance whilst mother finished school.  **N&CC**  Not discussed |  | **Parenting**  Father not discussed; Grandfather involved with childcare  **Love & care**  Closeness especially with grandfather;  **Grandparent Health**  Grandmother has dislocated hip | **Positive**  Grandparents do not drink alcohol in the home.  **Negative**  Provides sugar sweetened beverages |
| **Participant 13**  Grandmother  14 Grandchildren, 3 great grandchildren. Cares for grandson (7 years) & granddaughter (12 years).  After school one day p/w & sometimes overnight care at weekend | **H&PC**  Parents working  One daughter and partner worked shift work  One daughter in law works late shifts.  Providing free childcare allowed two of her daughters to buy their own homes.  Comments on parents not being well paid (Father gardener)  **N&CC**  Park | Responsible caregiving  Fun caregiving | **Parenting**  Father cohabits; Grandmother  comments that he suffers from alcoholism; Grandfather died  **Tensions**  Concern about role models; Father’s alcoholism; Unhealthy food given by mother; Lack of outdoor play  **Love & care**  Mutual respect between adults and children; Firm boundaries but also greater leniency; Love rather than discipline; Pride in grandchildren’s successes; Enduring relationships; happiness  **Grandparent Health**  Arthritis | **Positive**  Home cooked meals  Outdoor play/exercise  Limits takeaways  Encourages fruit intake  Encourages cooking  Limits foods HFSS  Walking  Limits screentime  Homemade food  **Negative**  Provides foods HFSS  Sweets as reward for good behaviour  Smoking |
| **Participant 15**  Mother (Daughter of Participant 16)  Four children (20 years, 16 years, 11 years & 18 months)  Grandmother cares for 11 year old granddaughter often,  Used to care for 20 year old as mother was 15 years when born & cohabited with grandmother.  **Participant 16**  Grandmother (Mother Participant 15)  12 grandchildren (including 4 great grandchildren) Helped care for all  Cares for 3 grandchildren and 2 grandchildren during school holidays | **H&PC**  Grandmother  not working  Mother working in supermarket (hoping to move to night shift)  Grandparent care allows for work to be financially viable for mother  **N&CC**  Park | Responsible caregiving  Fun caregiving | **Parenting**  Break-up of parents’ relationships discussed; Grandparents divorced.  **Tensions**  Discipline; Mother providing foods HFSS  **Love & care**  Admiration for grandmother; Chance to re-parent; Patience; Enjoyment  **Grandparent Health**  Has fibromyalgia – concern about keeping up; Providing space/boundaries for grandmother  to have a break | **Positive**  Outdoor exercise/play  Provides fruit  Limits foods HFSS  **Negative**  Provides foods HFSS |
| **Participant 17**  Grandmother  Granddaughter (13 years)  After school care & weekends | **H&PC**  Parents working  Grandmother  works  Grandmother care allows for work to be financially viable for mother  **N&CC**  (In past) Museum  Art Gallery  Park | Responsible caregiving  Fun caregiving | **Parenting**  Father cohabits; Grandfather helps with childcare  **Enjoyment**  Care keeps her going | **Positive**  Outdoor exercise/play  Enrichment activities  Toothbrushing  Discourages energy drinks  **Negative**  Provides foods HFSS |
| **Participant 18**  Grandmother  3 grandchildren granddaughters (13 years - lives away, 2 years), grandson (7 years)  Cares for younger children sometimes at weekend & at ad hoc times for appointments,  Cared for grandson Mon-Fri before school-age | **H&PC**  Mother stopped working Mon-Fri & now only weekends  Father works full time  Grandmother not working  Children have additional needs and grandmother recognises parents need support  **N&CC**  Library  Park | Responsible caregiving  Fun caregiving | **Parenting**  Father cohabits; Parents of grandchild who lives away divorced but still sees father regularly; Grandfather and grandmother separated  **Tensions**  Tension over discipline  **Love & care**  Love as reward; Grandchild and grandmother express their love for each other; Importance of one-on-one time; Bond  **Grandparent Health**  Exhaustion of caring; Health problems limit care – hysterectomy, breast cancer, osteoporosis, arthritis. | **Positive**  Outdoor exercise/play  Playgroup  Enrichment activities  Focused attention  Routine  **Negative**  Screentime  Provides foods HFSS |
| **Participant 19**  Grandmother  3 grandchildren – baby (<1 years), grandson (8 years) granddaughter (14 years)  After school care Mon-Fri | **H&PC**  Both parents working  Grandmother not working  Grandparent care allows parents to work full time and pay mortgage  Better lifestyle for parents than grandparents had | Responsible caregiving | **Parenting**  Father cohabits; Grandfather’s care contribution not discussed  **Tensions**  Less strict than parents and parents happy with discipline  **Love & care**  Intense love described; Different love from own children  **Enjoyment**  Grandchildren want to spend prolonged periods of time with grandmother | **Positive**  Encouraging sports  Not smoking around grandchildren  Allowing the children to try different fruit  **Negative**  Provides foods HFSS |
| **Participant 20**  Grandmother  Grandchildren: grandson (6 years & 10 years), and three older GC (15, 16 and 20 years)  After school care Mon-Fri, overnight stay Sat | **H&PC**  Both parents working  Grandparent care allows parents to work full time and pay mortgage  Better lifestyle for parents than grandparents had  Grandmother not working | Responsible caregiving | **Parenting**  Father cohabits  **Tensions**  Less strict than parents but parents happy with discipline  **Enjoyment**  More enjoyment caring for grandchildren | **Positive**  Outdoor exercise/play  Suncream for grandson with sensitive skin  **Negative**  Provides sweets |
| **Participant 21**  Mother  Daughter (6 years); Son (4 years)  Care – weekends ad hoc care | **H&PC**  Mother works part time in pub (previously worked night shift in casino), Grandparents not working  **N&CC**  Community centre activities - soft play, karate & gymnastics  Park  Art gallery | Responsible caregiving  Fun caregiving | **Parenting**  No father mentioned; Grandfather major role in childcare  **Tensions**  Thinks parents could discipline more but overall similar parenting approaches  **Love & care**  Described closeness of her children to grandparents  **Enjoyment**  Fun; Keeps them young  **Grandparent Health**  Parents have slowed down; Grandparents enable mother to engage in self care; Sees strain of childcare on grandparents due to age | **Positive**  Cooking with children  Homemade food  Enrichment activities  Encourage sport  Outdoor play/exercise  **Negative**  Smoking but not inside with children (though children witness it) |
| **Participant 22**  Mother  Daughter (8 years), Son (2 years), pregnant with 3^rd^  Grandmother cares for grandchildren in early mornings | **H&PC**  Works as student midwife (shift work), and money limited by bursary. Grandmother cares for the early morning. Mother couldn’t do course without grandmother  Child care prohibitively expensive  Grandmother also does shift work  **N&CC**  Summer holiday programme | Responsible caregiving | **Parenting**  Father cohabits; Grandmother lives with her husband (mother mentions Grandmother had been a single parent)  **Tensions**  Trusted caregiver  **Love & care**  Close relationship | **Positive**  Outdoor play/exercise  Some homecooking  Healthy snacks  **Negative**  Some processed foods  Screentime |
| **Participant 23**  Grandmother  2 grandsons (8 & 12 years)  Cohabiting  **Participant 24**  Mother  Daughter-in-law of Participant 23 | **H&PC**  Father working and mother works part time am  Cost of childcare prohibitively expensive. Few jobs available  Previously worked evening shifts for a bank with grandmother  providing care  Grandmother not working  **N&CC**  Activities too expensive | Not discussed | **Parenting**  Father cohabits; Grandfather died  **Tensions**  Grandparents greater leniency; Grandmother lets parents take overall responsibility for children  **Love & care**  Acts of care and love described  **Grandparent Health**  First grandson’s birth helped grandmother after grandfather’s death | **Positive**  Prepares homemade food  Grandparents helped reduce loneliness and isolation from wider community  **Negative**  Provides foods HFSS |
| **Participant 25**  Grandmother  Granddaughter (4 years)  Grandmother  provides adhoc childcare  **Participant 26**  Mother  Daughter-in-law of P25 | **H&PC**  Mother no longer working  Grandmother not working  Housing expensive and without a garden | Fun caregiving | **Parenting**  Father cohabits; Grandfather helps with childcare  **Tensions**  Greater leniency from grandmother  **Love & care**  Close relationship between granddaughter and grandmother | **Negative**  Provides food too often when granddaughter visits |
| **Participant 27**  Grandmother  7 grandchildren, grandson (7 years), granddaughters (9 & 17 years), plus 4 non cohabiting grandchildren  Grandchildren cohabit  **Participant 28**  Mother  Daughter-in-law of Participant 27 | **H&PC**  Mother not working  Grandmother not working | Responsible caregiving | **Parenting**  Father cohabits; Grandfather helps with childcare  **Tensions**  Similar approach to parenting as daughter-in-law  **Love & care**  Lots of love from grandchildren | **Positive**  Ensures grandchildren eat breakfast  **Negative**  Grandparents do not encourage children’s participation in sport |
| **Participant 29**  Grandmother  3 grandchildren (6 & 7 years – no age given for 3^rd^ grandchild).  Cohabiting and provides adhoc care  **Participant 30**  Mother  Daughter-in-law of Participant 29 | **H&PC**  Mother worked previously  Childcare prohibitively expensive – state funded doesn’t allow women to work  Grandmother not working | Responsible caregiving | **Parenting**  Father lives with children; Grandfather helps with childcare  **Love & care**  Close relationship between grandmother and grandchild; Importance of being a gentle carer to children – patience and care | **Positive**  Patient carer  **Negative**  Providing crisps to grandchildren |
